# Supplementary material for: Broadly neutralizing antibodies isolated from HEV convalescents confer protective effects in human liver-chimeric mice
Source: Nat Commun. 2025 Feb 26;16:1995. doi: 10.1038/s41467-025-57182-1 (PMC11865592; doi:10.1038/s41467-025-57182-1)

Supplementary Information

Broadly neutralizing antibodies isolated from HEV convalescents confer protective effects in human liver-chimeric mice

George Ssebyatika[#1], Katja Dinkelborg[#2,3,4], Luisa J. Ströh[5], Florian Hinte[6], Laura Corneillie[7], Lucas Hueffner[2], Elina M. Guzman[1], Prossie L. Nankya[1], Nina Plückebaum[5], Lukas Fehlau[2], Jonathan Garn[2], Nele Meyer[2], Sarah Prallet[8], Ann-Kathrin Mehnert[8], Anke R. M. Kraft[3,4,9], Lieven Verhoye[7], Carina Jacobsen[5], Eike Steinmann[10], Heiner Wedemeyer[3,4,11] , Abel Viejo-Borbolla[5,11], Viet Loan Dao Thi[4,8], Thomas Pietschmann[2,4,11], Marc Lütgehetmann[4,12], Philip Meuleman[7], Maura Dandri[4,6], Thomas Krey[1,4,5,11,13 *], and Patrick Behrendt [2,3,4*]

[1]Center of Structural and Cell Biology in Medicine, Institute of Biochemistry, University of Luebeck, Luebeck, Germany

[2]TWINCORE, Centre for Experimental and Clinical Infection Research, a joint venture between Helmholtz-Centre for Infection Research and Hannover Medical School, Hannover, Germany.

[3]Department of Gastroenterology, Hepatology, Infectious diseases and Endocrinology, Hannover Medical School, Hannover, Germany

[4]German Center for Infection Research (DZIF), Braunschweig, Germany

[5]Institute of Virology, Hannover Medical School, Hannover, Germany

[6]Department of Internal Medicine, University Medical Center Hamburg-Eppendorf, Hamburg, Germany.

[7]Laboratory of Liver Infectious Diseases, Department of Diagnostic Sciences, Faculty of Medicine and Health Sciences, Ghent University, Ghent, Belgium

[8]Schaller Research Group, Department of Infectious Diseases, Virology, University Hospital Heidelberg, Center for Integrative Infectious Diseases Research (CIID), 61920 Heidelberg, Germany.

[9]Centre for Individualised Infection Medicine (CiiM), a joint venture between Helmholtz-Centre for Infection Research and Hannover Medical School, Hannover, Germany
[10]Department of Molecular and Medical Virology, Ruhr University Bochum, Bochum, Germany
[11]Cluster of Excellence RESIST (EXC 2155), Hannover Medical School, Hannover, Germany
[12]University Medical Center Hamburg-Eppendorf, Institute of Medical Microbiology, Virology and Hygiene, Hamburg, Germany
[13]Centre for Structural Systems Biology (CSSB), Hamburg, Germany

Table of Contents:

Figure S1: Characterization of recombinant HEV P domains used in this study

Figure S2: FACS plots showing the gating strategy for the sorting of single HEV-specific memory B cells

Figure S3: Neutralization of HEV by single chain variable fragments (scFvs)

Figure S4: Kinetic binding analysis of bnAbs with pORF2 P-domain

Figure S5: Broad nAb reactivity with GT 3 strain 83-2

Figure S6: Immunofluorescence analysis using human α-pORF2 IgGs

Figure S7: Epitopes targeted by glycan-sensitive human bnAbs

Figure S8: Electron density maps for HEV bnAbs in complex with P domain

Figure S9: Semiquantitative analysis of glycan-sensitive Abs in patient sera

Figure S10: nAb p60.1 prevents fecal-oral HEV infection in human liver-chimeric mice

Figure S11: Amino acid alignment of P domain sequences showing conservation and residues involved in interaction with bnAbs p60.1 and p60.12

Table S1: Sequence information of HEV pORF2 P-domain-specific antibodies

Table S2: Kinetic interaction analysis between bnAbs and glycosylated and non-glycosylated GT 3 P domains

Table S3: Characteristics of patient samples used for ELISA and isolate neutralization in HLCs

Table S4: Kinetic interaction analysis between bnAbs and non-glycosylated GT 3 P domains of different strains

Table S5: Diffraction data collection and refinement statistics

Table S6: Accession numbers of virus strains and isolates used in this study

Table S7: HEV antigen detection by different ELISAs

Table S8: List of oligonucleotides used in this study

Source Data for Figure S1

Figure S1: Recombinant HEV P domains used in this study

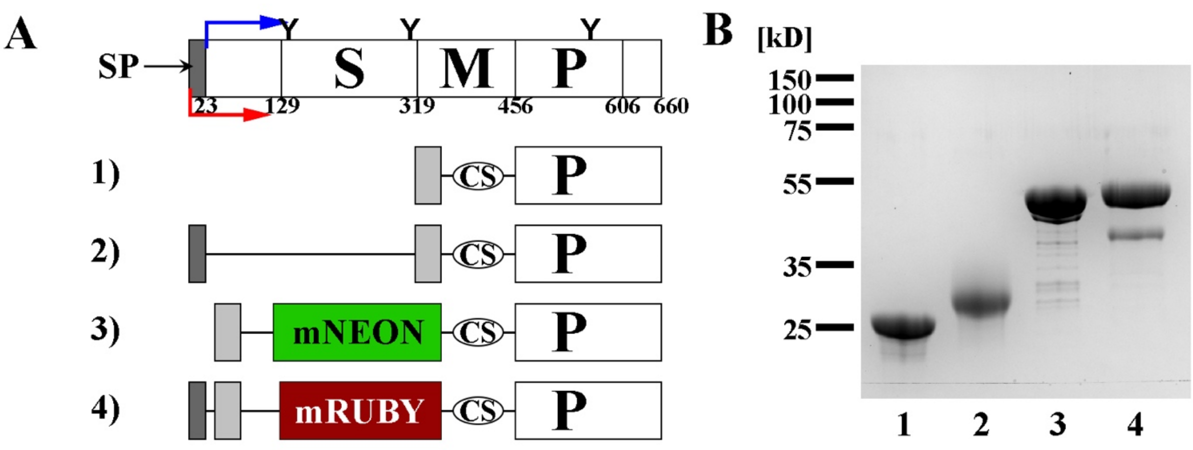

Figure S1: A) Schematic primary structure of ORF2, its individual domains (S/M/P), and the different expression constructs for the recombinant GT 3 P domains used in this study. Red and blue arrows indicate the described start codons for the glycosylated and non-glycosylated ORF2 in the presence and absence of a signal peptide, respectively. Numbers indicate amino acid positions within the GT 3 ORF2. SP, signal peptide (dark grey box); DST, double strep tag (light grey box); CS, proteolytic cleavage site for removal of affinity tag and fluorophore. Possible N-linked glycosylation sites are indicated. B) SDS-PAGE analysis followed by Coomassie staining reveals a slower migration of the glycosylated P domain (lanes 2 and 4) compared to the non-glycosylated P domain (lanes 1 and 3), likely caused by the attached glycan at position 562. Lanes 1-4 correspond to constructs 1) - 4) in panel A. Source data are provided within the Source Data file.

Figure S2: FACS plots showing the gating strategy for the sorting of single HEV-specific memory B cells

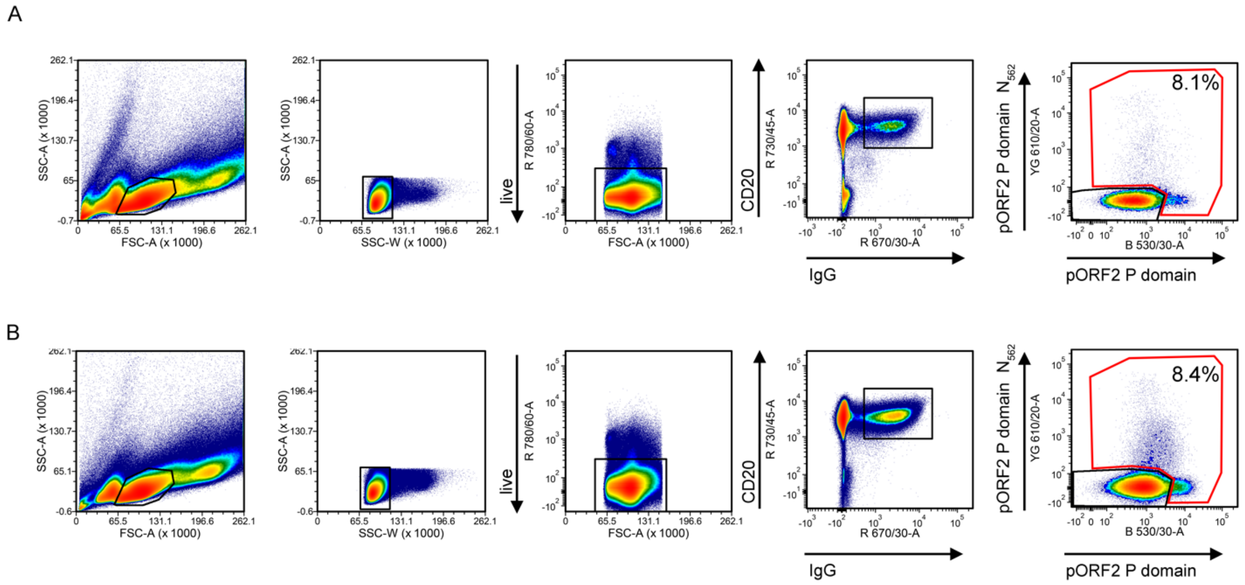

Figure S2: Representative FACS plots showing the gating strategy for sorting single pORF2 P-domain-specific memory B cells. (A) patient p60 and (B) patient p61. The red gate highlights the sorted cells.

Figure S3: Neutralization of HEV by single chain variable fragments (scFvs)

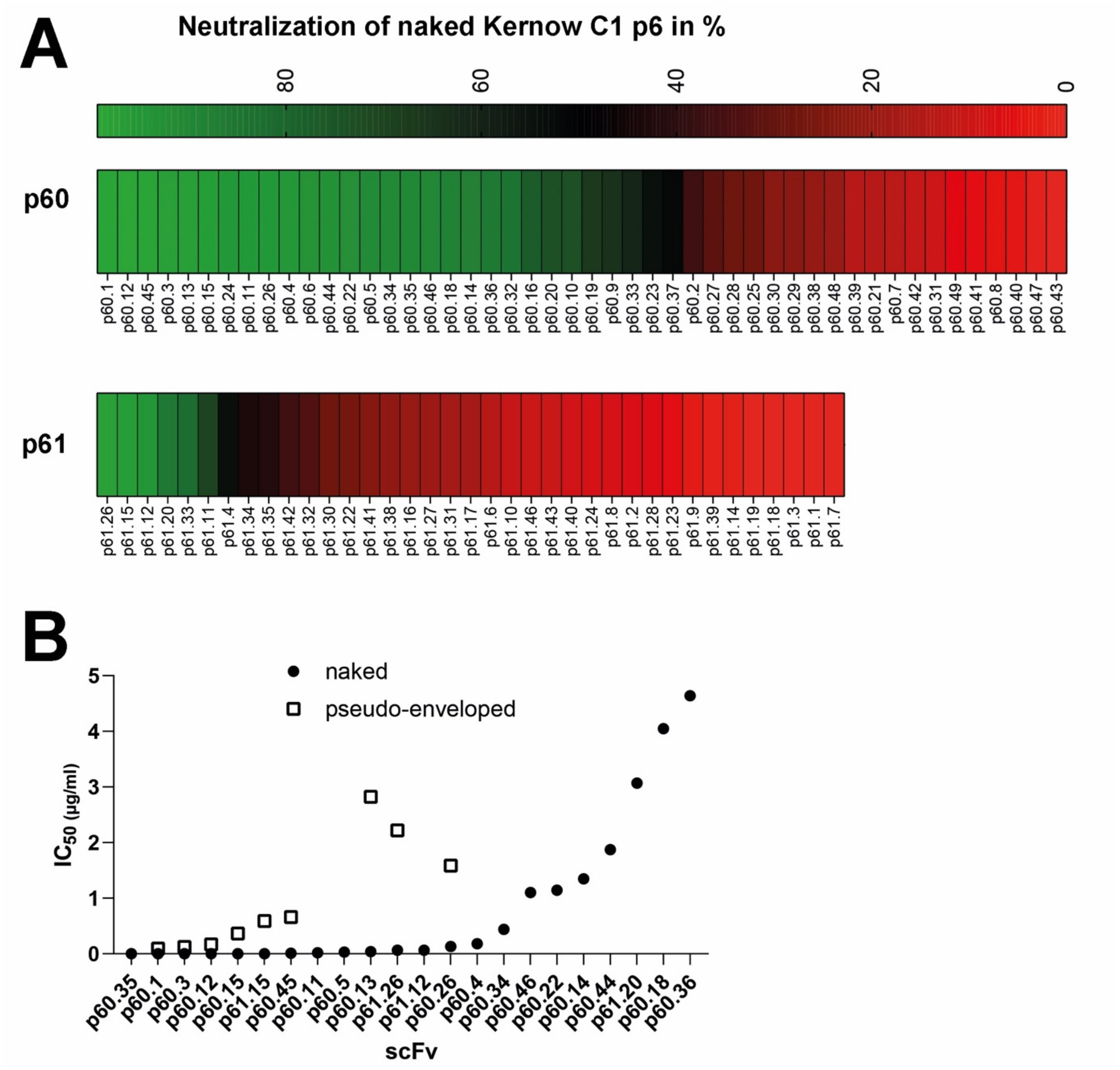

Figure S3: (A) 85 scFv expressed from patients 60 (p60) and 61 (p61) ranked according to their neutralizing capacity at a concentration of 10 µg/ml, with samples colored green showing at least 50% neutralization. Values are normalized to the control in the absence of scFv (n= 2 biological replicates) (B) The best neutralizers from (A) were further tested in serial dilution at concentrations of 5, 0.5, 0.05, and 0.005 µg/ml against naked HEV (dots) and at concentrations of 10, 5, 0.5, and 0.05 µg/ml against pseudo-enveloped HEV (boxes) to calculate the IC_{50} using GraphPad Prism 9. Samples are ranked for the IC_{50} against naked HEV. Neutralization of pseudo-enveloped HEV was not observed for all tested scFvs. Source data are provided within the Source Data file.

Figure S4: Kinetic binding analysis of bnAbs with pORF2 P-domain

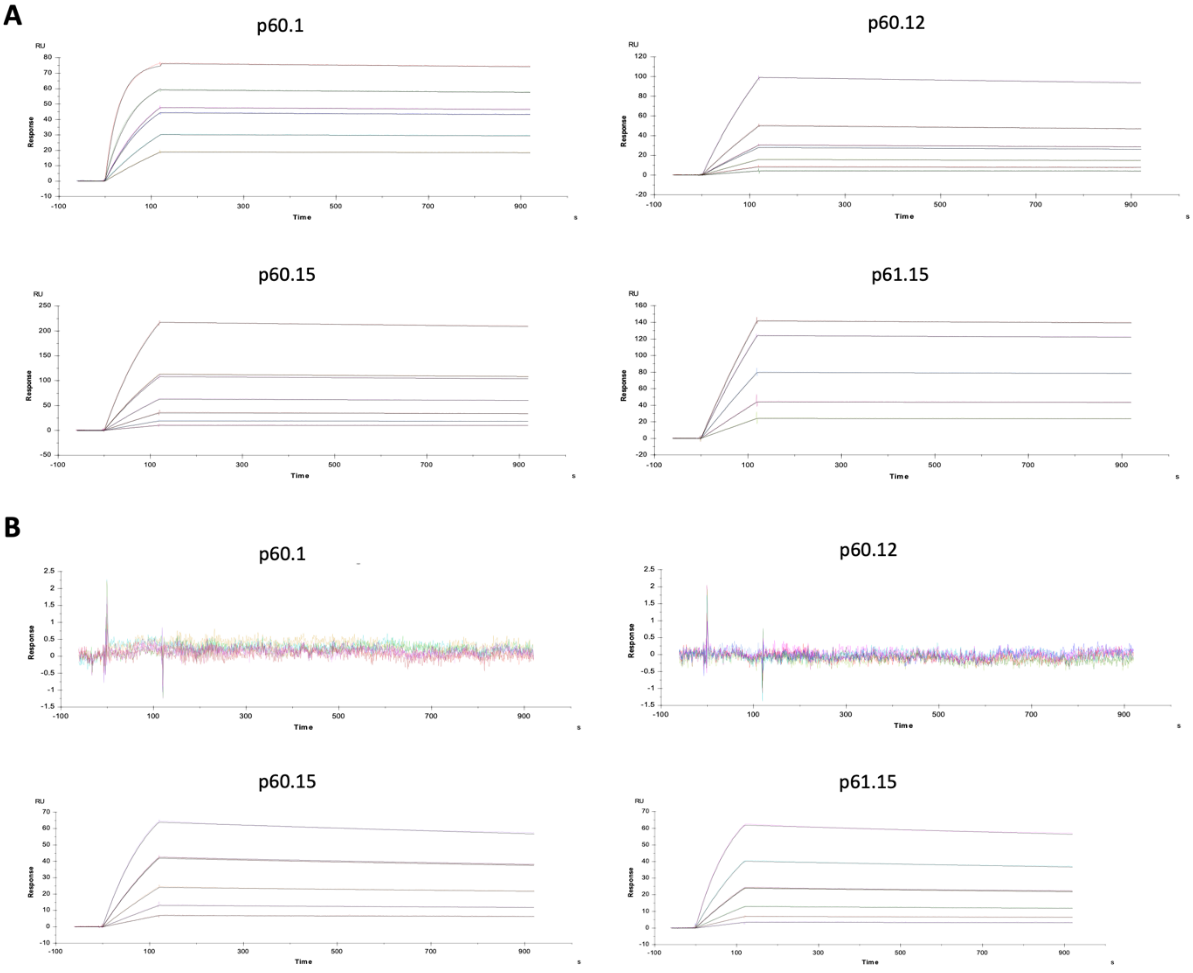

Figure S4: SPR analysis of nAbs p60.1, p60.12, p60.15, and p61.15 at 25, 12.5, 6.25, 3.125, and 1.5625 nM to the immobilized non-glycosylated (A) or glycosylated (B) HEV GT 3 P domain. The colored lines represent a 1:1 kinetic model fit. The kinetic binding parameters are shown in Table S2.

Figure S5: Broad nAb reactivity with GT 3 strain 83-2

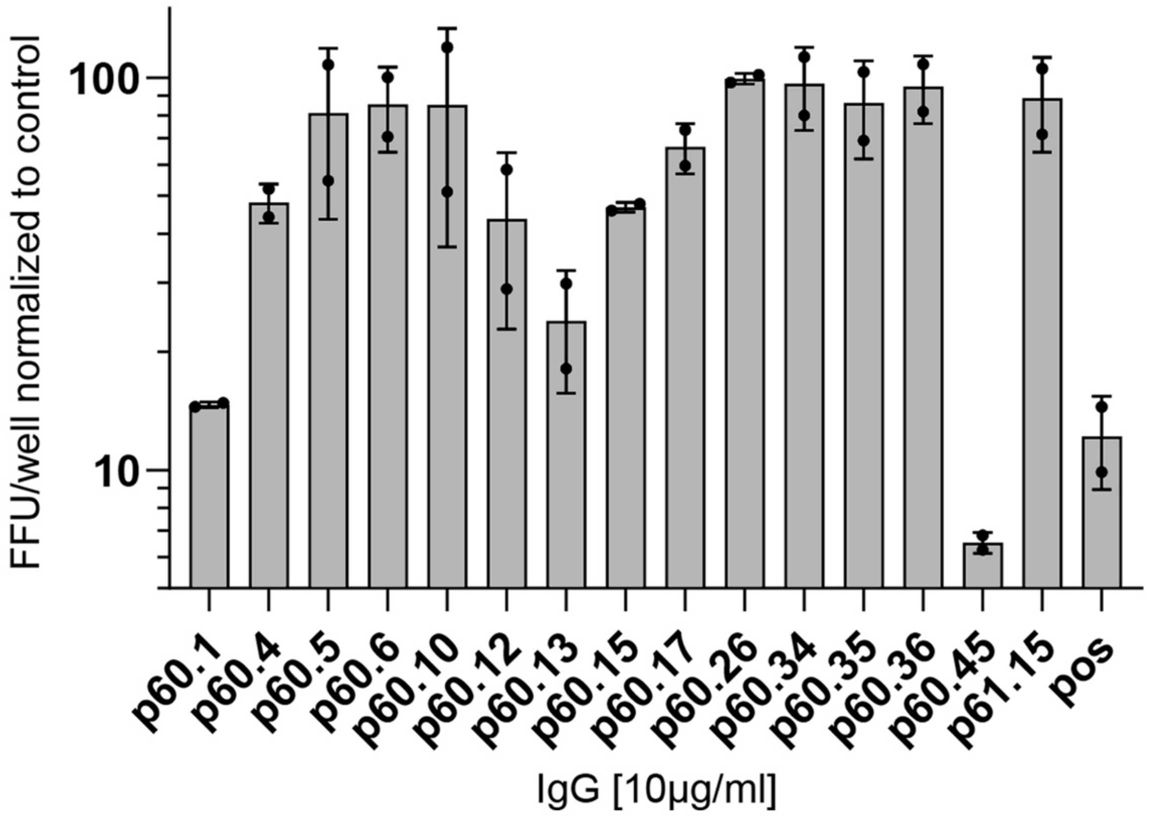

Figure S5: Percent of focus forming units per well of HepG2/C3A cells infected with HEV pUC83-2-27 neutralized with the indicated antibodies at a concentration of 10 µg/ml. The values are normalized to the control, to which no antibody was added. A human anti-HEV positive serum was used as positive control (pos) (n= 2 biological replicates). Source data are provided within the Source Data file.

Figure S6: Immunofluorescence analysis using human α-pORF2 IgGs

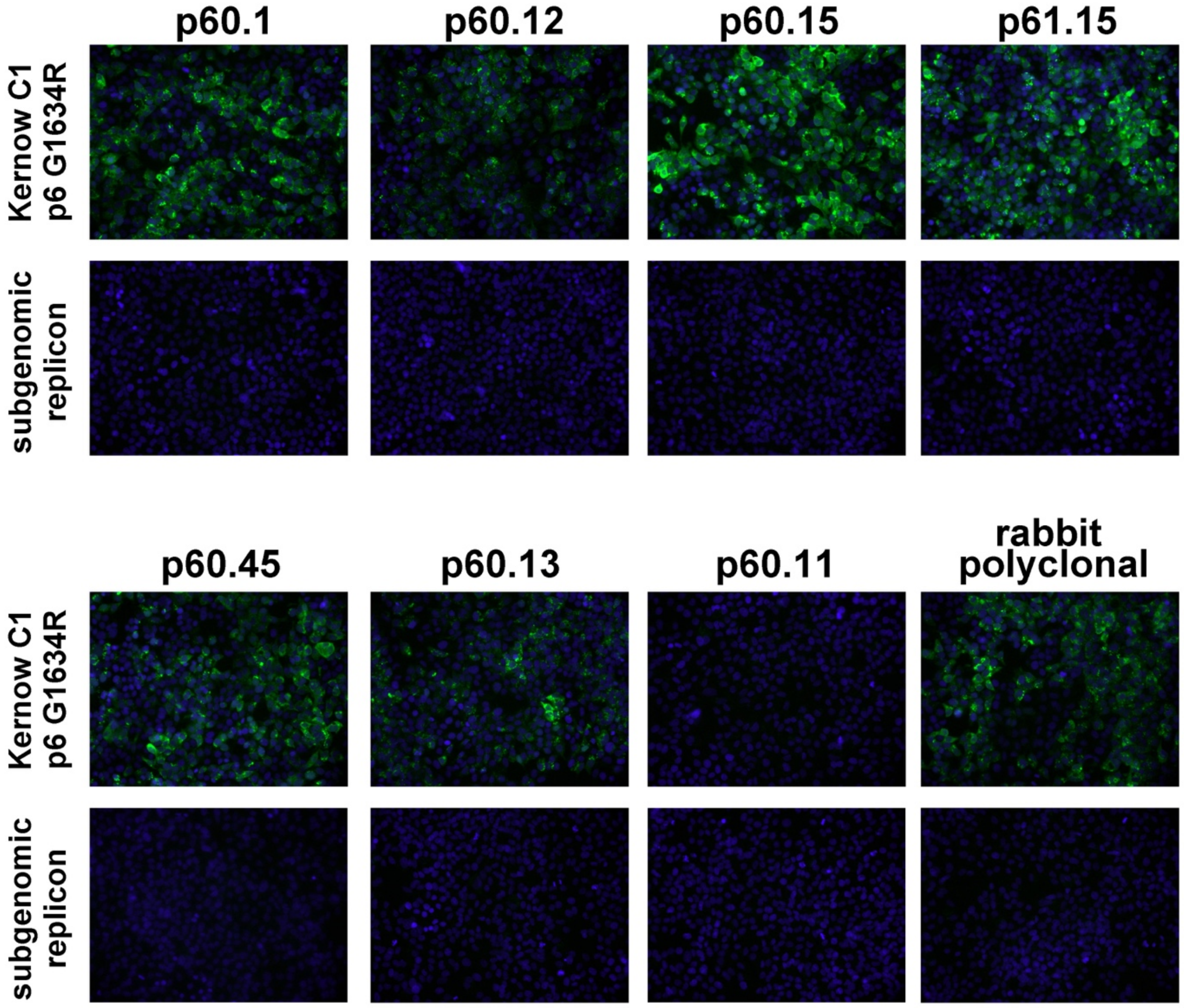

Figure S6: Immunofluorescence images depict HepG2 cells transfected with the viral genome (Kernow C1 p6 G1634R) or a subgenomic replicon lacking ORF-2 as control (subgenomic replicon). Four days after transfection, cells were washed and fixed using 3% PFA and permeabilized with Triton X-100 prior to staining with 0.25 µg/ml of the indicated primary antibodies. As control, a rabbit polyclonal anti-P domain antibody was used. Bound nAbs were detected using fluorescently labelled anti-human or anti-rabbit secondary antibodies. Images were taken at 20x magnification, and nuclei stained with DAPI. The experiments were repeated three times with similar results.

Figure S7: Glycan-sensitive antibodies bind to a conserved epitope at the tip of the P domain dimer

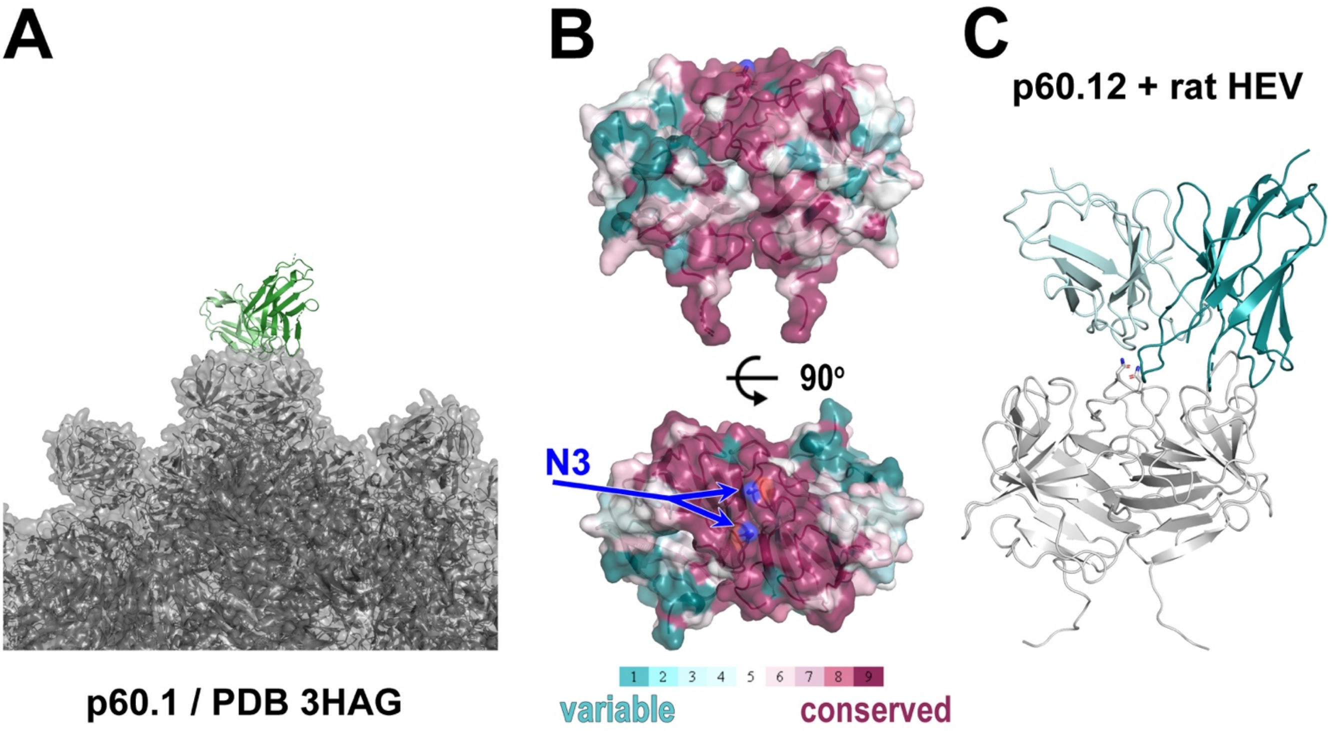

Figure S7: A) Superposition of the crystal structure of the P domain in complex with bnAb p60.1 to the crystal structure of HEV virus-like particles (PDB 3HAG). The VLP is shown as surface representation in grey, while p60.1 is shown as a green-coloured cartoon. B) Amino acid conservation of different HEV genotypes used in this study (Table S4) mapped onto the P domain crystal structure. Sidechain oxygen and nitrogen atoms of N562 are colored in red and blue, respectively, and a blue arrow indicates the position of N3. Amino acid sequence alignments were performed using Clustal Omega (EBI), and the conservation was mapped using the ConSurfDB server (https://consurfdb.tau.ac.il/). C) Cartoon view of the complex crystal structure of the rat HEV P domain (grey) and bnAb p60.12 (cyan). Sidechain oxygen and nitrogen atoms of N_{562} are colored in red and blue, respectively, to indicate the position of N3.

Figure S8: Electron density maps for HEV bnAbs in complex with P domain

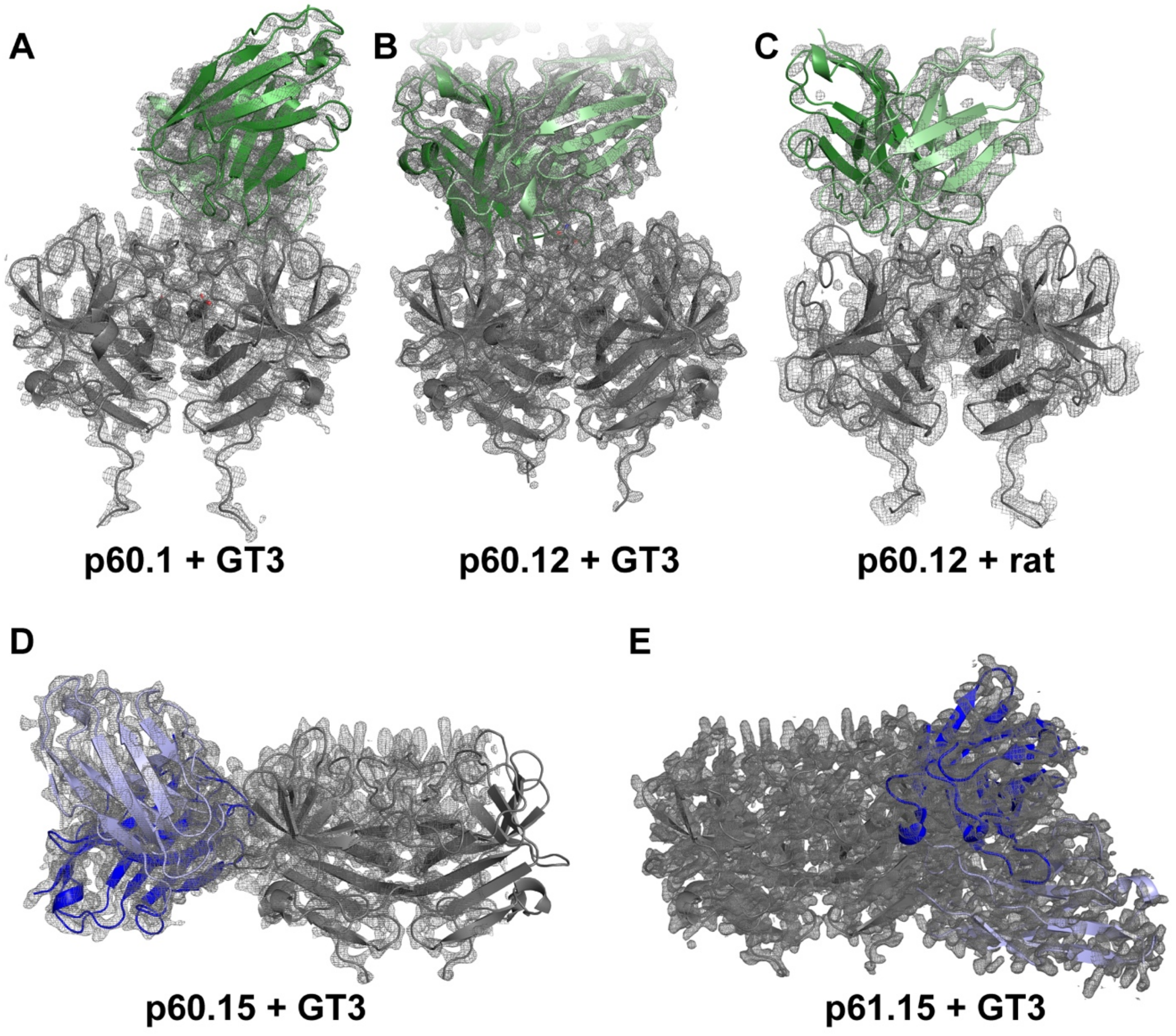

Figure S8: 2Fo-Fc electron density maps contoured at 1.5 σ are superimposed on the corresponding refined atomic models shown as cartoon. For clarity, one P domain dimer is shown in each case. A GT 3 strain P domain was crystallized in complex with (A) p60.1, (B) p60.12, (D) p60.15, and (E) p61.15. (C) A rat HEV P domain was crystallized in complex with p60.12.

Figure S9: Glycan-sensitive Abs are induced during acute HEV infection

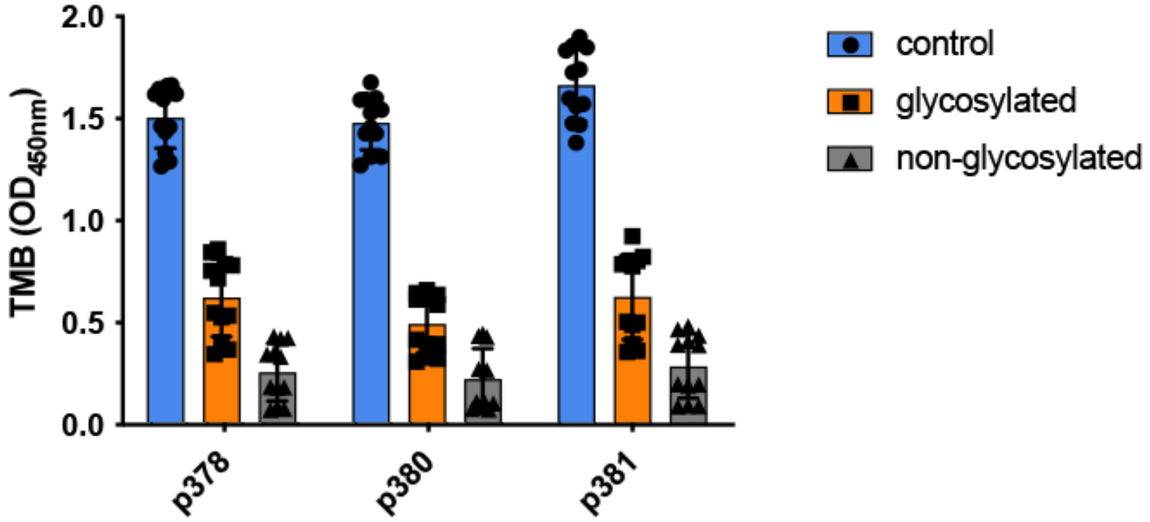

Figure S9: Sera from three patients, who underwent an acute HEV infection (p378, p380, and p381) were depleted in parallel with either glycosylated (orange), non-glycosylated (grey) or no (control, blue) GT3 P domain and subsequently subjected to a direct ELISA against the non-glycosylated P domain. The signal difference between the two differentially depleted serum samples is likely caused by steric interference of the glycan chain attached to N_{562} with antibodies recognizing a juxtaposed epitope. The chart shows the mean of four independent biological replicates, error bars indicate the standard deviation. Source data are provided within the Source Data file.

Figure S10: nAb p60.1 prevents fecal-oral HEV infection in human liver-chimeric mice

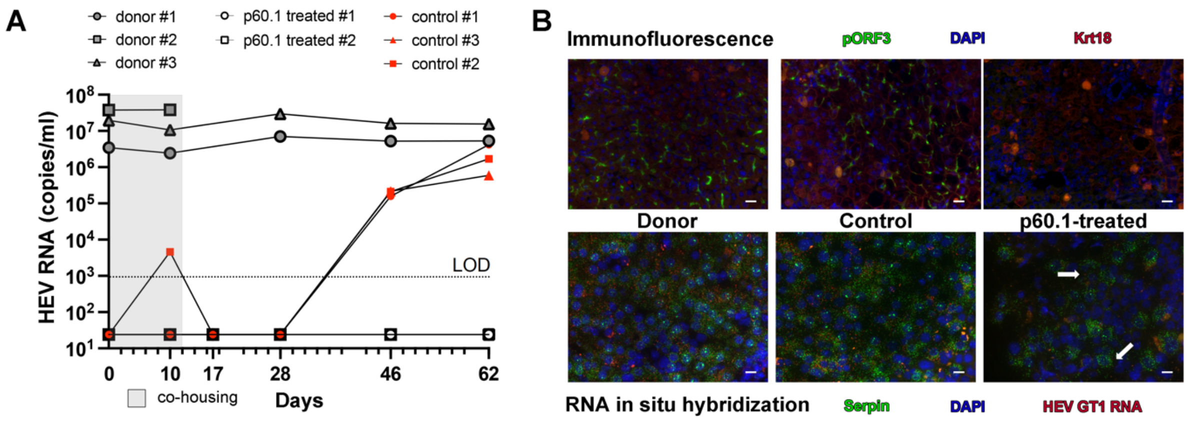

Figure S10: (A) Viral titers in the serum (copies/ml) of three donor mice (grey symbols), two antibody-treated mice (red symbols), and three control antibody-treated mice (empty symbols) were monitored over 62 days. At specific time points, blood was taken retrobulbar, viral RNA was isolated and measured by quantitative RT-PCR. LOD = 1000 copies/ml, light grey box = co-housing phase. Source data are provided within the Source Data file. (B) Visualization of HEV GT1 ORF3 protein (pORF3) in the liver of infected mice. Representative immunofluorescent staining of pORF3 (green) in liver sections from donor mice, control antibody-treated mice, and p60.1-treated mice. Human hepatocytes are stained with anti-Krt18 antibodies (red), and nuclei are visualized with DAPI (blue). Scale bars represent 20 µm.

Figure S11: Amino acid alignment of P domain sequences showing conservation and residues involved in interaction with bnAbs p60.1 and p60.12

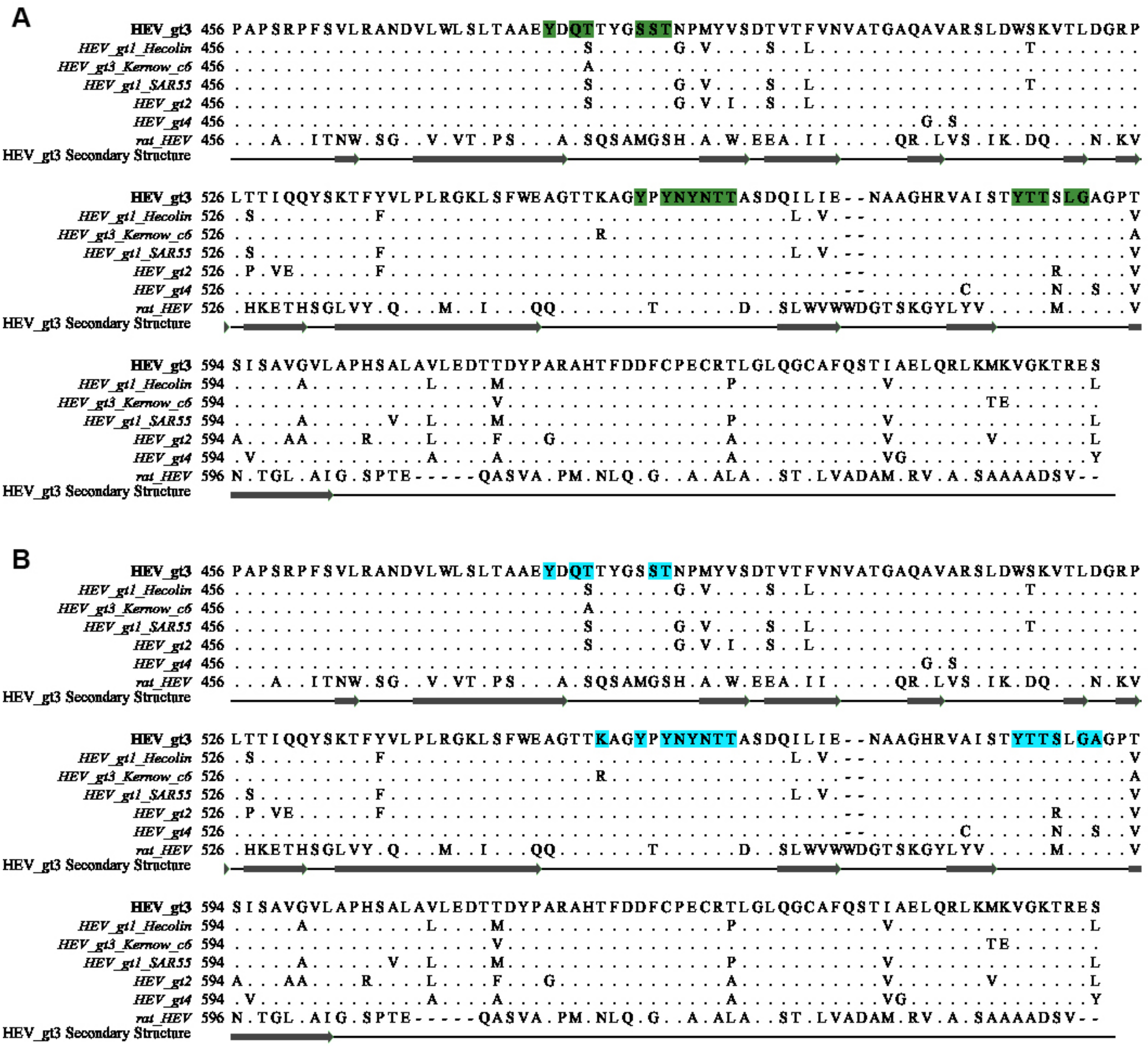

Figure S11: Amino acid alignment of the P domain sequences used in this study and the Hecolin® sequence. The secondary structure elements of the P domain are shown below the alignment. Residues involved in interaction with bnAb p60.1 (A) are colored in green, residues interacting with p60.12 (B) in cyan. Dots represent conserved amino acid residues. Amino acids numbered according to HEV genotype 3 (UniProt accession number C4B4T9)

Table S1: Sequence information of HEV pORF2 P-domain-specific antibodies

Ab code	Donor	VH germline gene	CDRH1	CDRH2	CDRH3	VL germline gene	CDRL1	CDRL2	CDRL3
p60.1	p60	IGHV 6-1	GDSVSTINAA	TYYRSKWYH	CARDRGPTPFDFW	IGKV 1-39	QSISRS	AAS	CQQSFVTPFTF
p60.2	p60	IGHV 6-1	GDSVSTINAA	TYYRSKWYH	CARDRGPTPFDFW	IGLV3-21	NIGSKS	DDS	CQVWDSSSDQGVF
p60.3	p60	IGHV 6-1	DSVSNINVA	TYYRDKWYT	CASHGGSDPYAFDIW	IGLV 1-40	RSNIGTRYS	GNI	CQSYDISLSGYVF
p60.4	p60	IGHV 1-2	GYTFTGIS	INPKTGGT	CARLDLLWSGELTGIPKIYYYYGMDVW	IGKV 1D-39	QSISNY	AAS	CQQSDSTPFTF
p60.5	p60	IGHV 3-21	GFTFSSYS	ISSSSSYI	CARGGGLMITFGGVMGGMDVW	IGKV 2-28	QSLLHSNGYNY	LGS	CMQALQTPYTF
p60.6	p60	IGHV 3-23	GFTFSSYT	MSGSGDNT	CATRRLYCTTTSCYMYYFDDW	IGLV 2-8	STDVGGYNY	DVN	CSSYAASDTLLF
p60.7	p60	IGHV3-30	GFPFSTYG	ISYDGNNK	CAKDDYASGPFFDYW	IGKV1-9	QGISSY	AAS	CQQLNTYPITF
p60.8	p60	IGHV4-30-2	GSSIRSGDYY	IYYSGTT	CARAGRKTTVVTPDLDYW	IGLV1-44	WTNIGDNY	KHS	CASWDDSLRGWVF
p60.9	p60	IGHV5-51	GYSFTSYW	IYPGDSDT	CARSRGDRGFFDPW	IGKV3-20	QSVSSTY	GAS	CQHYGSSPHTF
p60.10	p60	IGHV 6-1	GDSVSSNSAA	TYYRSKWYN	CASGSYQHFDYW	IGLV 2-23	SSDVGSYNL	EVS	CCSYAGSSTYYVF
p60.11	p60	IGHV 1-46	GYTFTSYY	INPSAGHT	CARAALRRREILDYW	IGLV 3-21	NIGSRS	DGS	CQMWDTSSDRGVF
p60.12	p60	IGHV 1-69	GDTFSSYV	IIPIIGTA	CASNVQLQRRGNWFDPW	IGLV 2-14	SSDIGDYNF	DVT	CSSYTSTNTPVVF
p60.13	p60	IGHV 3-48	GFSFRSYE	ITSSGSGT	CATVIRRDGYNSDW	IGLV 1-40	TSNIGAPYD	GNT	CQSYDNSLSGSVF
p60.14	p60	IGHV 4-4	GGSISSNNW	IHHSGST	CAREAIYYNGMDVW	IGKV 3-20	QRISSRY	GAS	CQQYASSPKTF
p60.15	p60	IGHV 5-51	GYDFANYW	IYPRDSDI	CARHLRGLRLGELFPFDYW	IGKV 1-9	QDIFSY	AAS	CQQLNRYPFAF
p60.16	p60	IGHV1-18	GYSFTSYG	ISGNNGNP	CARHLDYYDSSGYYPRWYFDFW	IGLV3-21	SIGGKN	DDS	CQVWDTDTDHVIF
p60.18	p60	IGHV 1-18	GFPFTNYG	ITPYNRNT	CASLKRHGYNLPYFDYW	IGKV 1-12	QGVNSW	AAS	CQQANSFPYTF
p60.19	p60	IGHV 1-18	GYTFTSYG	ISVHNGNI	CARDGGWNSRLFDYW	IGLV1-44	NSNVGTIA	SNN	CATWNDSLNDWVF
p60.20	p60	IGHV1-18	GYTFTSYG	ISAYNGNT	CARDRGSFIGSISGVADEFDNW	IGKV2-30	QGLVYIDGNTY	KVS	CMQGTHWPYTF
p60.21	p60	IGHV 1-18	GYTFTSYG	ISGYNGNT	CAREGSSGWHRLIDYW	IGLV1-44	SSNIGTKT	TNN	CAAWDDSLNGWVF
p60.22	p60	IGHV 1-18	GYTFATYD	ISTYSGNT	CARDDKWRSITPSYSSHGYAPLDYW	IGKV 1-5	QSISSW	KAS	CQQYSSYSPTF
p60.23	p60	IGHV1-18	GYTFTAYG	INGYNGNT	CARVGTYDTSGYYWLW	IGKV1-8	QDIGSF	AAS	CQQYDSYPRTF
p60.24	p60	IGHV 1-18	GYTFSSYA	ISAYNGNT	CARYYYYGWGGFDPW	IGKV 3-20	QSVSSSSY	GAS	CQQYGSSPYTF
p60.25	p60	IGHV 1-18	GYTFSSYG	ISAYNGDS	CARDRHSHYDIRLGHYHYAMDVW	IGLV3-19	SLRSYY	GEN	CNSRDSSGNLLGIF
p60.26	p60	IGHV 1-18	GYNFTTYS	ISNYNGKA	CARDPIYDSGSYIAHFQYYGIDVW	IGLV 3-25	ALPKQY	KDS	CQSADSSGSWVF
p60.27	p60	IGHV 1-18	GYIFTSFG	ISTYNGNT	CARDKVPRSGHRAYYHYGMEVW	IGLV2-8	SSDVGGYNY	EVS	CSSYVSNNNLVF
p60.28	p60	IGHV 1-18	GYTFSSYG	ITPYNGNT	CAGSVEVERDYYYYGMDVW	IGKV1D-39	QSISTY	AAS	CQQSYSTPPSTF
p60.29	p60	IGHV1-18	GYNFHNYG	INTFTGNR	CARDVRCSGDSCDSYHFYGMDVW	IGKV3D-15	QSISSN	GAS	CQQYNNWPPWTF
p60.30	p60	IGHV1-18	GYTFTSYG	IRAVTGENVY	CARDQKEIVVVPAEYGMDVW	IGLV1-51	SSNIGNHF	DNN	CGTWDSSLRIVVF
p60.31	p60	IGHV1-2	GYRFTGYF	IDPDSGGT	CARGQDHVSYDVSLGDHW	IGLV1-51	STNIGDNY	ENN	CGTWDRSLSAWVF
p60.32	p60	IGHV1-2	GYSFTGTY	INPNSGGT	CAGGVGTTISDYW	IGLV2-11	SSDVGGYNY	DVT	CCSYAGSWVF
p60.33	p60	IGHV1-2	GFTFTGYY	ISPNSGGT	CARGRVVVPAVILYW	IGLV6-57	SGDISSNY	EDN	CQSYDSTSPWVF
p60.34	p60	IGHV 1-2	GYTFTGY	INPNSGGT	CLRGHYYDSSGLW	IGLV 4-69	SGHSSYA	LNSDGSH	CQTWGTGTGVF
p60.35	p60	IGHV 1-2	GYTFTGYY	INPNSGVT	CAKRAPIIPWVDPW	IGLV 6-57	SGSIASNY	EDD	CQSYDSDTVVF
p60.36	p60	IGHV 1-2	GYNFIGYY	INPNRGST	CARDIDTGSNSNDWFDPW	IGLV 3-10	ALPKKF	EDT	CYSTDSSGNHGGVF
p60.37	p60	IGHV 1-2	GYTFTAYY	INPNSGGT	CARWVVPGLYSMDVW	IGLV3-21	NIGSKR	DES	CQVWDSTTDPVIF
p60.38	p60	IGHV1-2	GYPFNVHY	ISPNTGGT	CAREGRGLTTMSRGITGYGMDVW	IGKV3-11	QSVSDY	DAS	CQQRSNWPLTF
p60.39	p60	IGHV1-24	GYTLTELS	FDPEDGER	CSTASLIAPIAWPFDYW	IGKV1D-39	QSISRH	GAD	CQQSYNIPWTF
p60.40	p60	IGHV1-24	GHTLPDLF	FDHEDGER	CATGIVEDLPGGLDYGLDVW	IGLV1-44	SSNVEFNS	AND	CSVWDDNLHVVF
p60.41	p60	IGHV1-3	GYMFSTHS	INTDTGNT	CARDRYYGSGHYNYFDYW	IGKV3-20	QAIDSKY	GIS	CQQYSSSPWTF
p60.42	p60	IGHV1-3	GYTFTTYS	INAGNGNT	CAREMPRIVGATGWFDPW	IGLV3-19	SLRSYY	GKN	CSSRDSSVNHPVLF
p60.43	p60	IGHV1-3	GYNLTDYA	VNGDNGDT	CAREGGDCTGTTCYGSYGMDVW	IGLV3-25	ALPKQY	KDS	CQSPDSSATYVF
p60.44	p60	IGHV 1-3	GYSFTNYA	INAGNGDT	CARVYSTSKTPRRDYSYYGMDVW	IGLV 1-40	SSNIGAGYD	GNN	CQSFDSSLSGWVF
p60.45	p60	IGHV 1-46	GYTFTSYF	INPSAGST	CARDLKTSGWQRQFDSW	IGLV 3-21	NIGGER	DDS	CQVWDSSSDQGVF
p60.46	p60	IGHV 1-46	GYTFTNYY	INPSGGST	CARDQAGSGYFDFW	IGKV 1-5	QSISSW	KAS	CQQFNSYPWTF
p60.47	p60	IGHV1-46	GYTFASYY	ISPGDNST	CARSPPPPSEISYSHLHFDYW	IGLV1-40	SNIGAGYH	DND	CQSYDSRLSGWVF
p60.48	p60	IGHV1-46	GYTFTSYY	INPSGGST	CARGNIVVVPAASNWFDPW	IGLV2-14	SSDIGAYNY	DVI	CSSYTTTRTLVF
p60.49	p60	IGHV1-58	GFPLNNSA	IVVGSGYT	CAADPVITIFGVFSPW	IGKV3-11	QNVHSF	DAS	CQQRTSWPRAF
p61.1	p61	IGHV1-2	GHTFTGYY	INPNSGGT	CARLYRDAFDIW	IGLV2-8	SSDVGGYNY	EVK	CASYAGSSSYVF
p61.2	p61	IGHV4-59	GGSFTDYY	INHKGNT	CARHFSVSALEFLTVVISRMGAFDVW	IGLV3-19	SLRKYY	GKD	CNSGDSNTYVF
p61.3	p61	IGHV2-5	GFSLRNSGLG	IYWDDDK	CAHSRRTVAGAFAPW	IGKV2-28	QSLLHSDGYNY	LGS	CMQGLQIPQTF
p61.4	p61	IGHV3-23	GFSFSSYA	ITGSGDTT	CAKVDVLMVSAIYYYAMDVW	IGKV1D-39	QSISSY	AAS	CQQSFRTSSYTF
p61.6	p61	IGHV3-74	GFTFRSDW	IRDDGSTS	CAREAPGRGNWYIDLW	IGLV3-25	ALPNEY	KDT	CQSADSSATWVF
p61.7	p61	IGHV1-2	GYTFTAYF	INAHSGGT	CARGEEFCSGGLCYFDSW	IGKV1-5	QRIRDY	KAS	CQQYDVYPISF
p61.8	p61	IGHV2-5	GFSLSTYGEG	IFWDDEK	CAHRRWIGPVTPDPFDIW	IGLV3-19	SLRHFS	DEN	CMSRDPTDHHRIF
p61.9	p61	IGHV3-23	GFTFSSYA	ISGSGDST	CAKAWTYYEYFFDYW	IGKV1-5	QSISSR	KVS	CQQYDTYSSF
p61.10	p61	IGHV4-59	GGSISSFY	VDYSGST	CARDASIIAPVGQDNWFDPW	IGKV1D-39	QTIRTS	AAS	CQQSSSIPITF
p61.11	p61	IGHV4-61	GASIGSGGYY	IFHSGST	CARRDCSSASCYFDYW	IGLV3-21	DIVRRS	DDS	CQVWDSTSDHPVVF
p61.12	p61	IGHV 3-23	GFTFSSYA	IDASAFGT	CAKSAMLRGHNVDYW	IGKV 1-16	QGINNY	AAS	CQQYRSYPVTF
p61.14	p61	IGHV4-34	SGFFTGFF	IDRGGTS	CARGRRIRGFDSW	IGKV1-17	QGIRND	SAS	CLQYNDYPYTF
p61.15	p61	IGHV 1-46	GYTFTNYF	FNPRGGST	CARMGSVTGPVGHRWLDPW	IGKV 3-11	QSVDTY	DAS	CQQRYNWPPITF
p61.16	p61	IGHV3-7	GFGFSNFW	IKDDGSEK	CMRGHYSNDW	IGKV1-27	QDIRH	DAS	CQKYNSVPWTF
p61.17	p61	IGHV4-39	GGSIRTHFYY	IFYSGKT	CARHPAAEIVGW	IGKV3-11	QSVGTY	DAS	CQQRTNWLFSF
p61.18	p61	IGHV1-2	GYSFSGYY	INPSSGDT	CARLTFSTGNFFSDRGWFDPW	IGLV1-47	TSNIAQYN	KTN	CASFDDSLSGWVF
p61.19	p61	IGHV1-46	GDTFASYN	FNPNPLIT	CAREETSGWVQW	IGLV6-57	SGNIDSNF	EDD	CQSYDGANQWIF
p61.20	p61	IGHV 1-8	GYSFTNFE	MNPNTGNT	CAREVGSGYYWFDPW	IGLV 3-19	SLRSYY	GKN	CSSRDTSGHYWVF
p61.22	p61	IGHV3-11	GFRFSDYY	ISERGHTI	CARTEEYGTNSRRGAFDSW	IGLV3-1	YLGNKY	QNY	CQAWDITTQYVF
p61.23	p61	IGHV3-15	GFTFSHAW	IKSKTDGGTT	CTTGAAQWLTTFDYW	IGKV1D-39	QTITTY	AAS	CQQTYSSPPVTF
p61.24	p61	IGHV3-30	GFTFTSYG	TSPDGTVN	CVKEIAFYDGYDIW	IGLV2-14	SSDVGGHNY	GVS	CSSYTSSGLYVF
p61.26	p61	IGHV 3-33	GFNFTRYA	LSYDGSDK	CARDPRRLLPMFYFDFW	IGKV 3-20	QSITSTY	DVS	CQHYGSSFLTTF
p61.27	p61	IGHV3-66	GFTVSTNY	NYSGGSI	CARAVVQLQSHYFDYW	IGKV3-20	QSVSSSY	GAS	CQQYGSSPATF
p61.28	p61	IGHV3-74	GFTFSGYW	INIDGSST	CVRDNWMSYW	IGKV2D-29	QSLLHSNGKTY	EVS	CMQSIQLPLTF
p61.30	p61	IGHV4-34	GGSFSGYY	IDHTGST	CARGGDFDSTTYHLFTW	IGLV3-21	DIGTKT	DDS	CQVWDTSSAQVVF
p61.31	p61	IGHV4-59	SGSISRYH	IYDDGST	CARVTGQKFFRAGMDYW	IGKV1D-39	QYIMKY	GAS	CQQSYSTPDTF
p61.32	p61	IGHV4-59	GGSISNYY	IYTREST	CATSATYGMEHW	IGKV1-9	HDISTY	LAS	CQQLRSYPLTF
p61.33	p61	IGHV5-51	GYTFRDYW	IFPGDSDT	CARRFYESGVDKYYFDLW	IGLV3-21	DVGSKA	DDN	CQVWDDNSDHFVF
p61.34	p61	IGHV1-18	GYIFTSYG	ISEYNGNT	CARDWGVAPLVPDYW	IGKV1-17	QGIRND	AAS	CLQHNTYPLTF
p61.35	p61	IGHV1-2	GYTFIGYY	INPNSGAT	CARDPVDTAIYPHYYFHYGLDVW	IGLV2-14	SSDVGGYNY	DVS	CSSYRGSSTLVVF
p61.38	p61	IGHV2-5	GFSLSASGVG	IYWDDEK	CAHRDYGDALQYW	IGLV1-51	DSNIGNNF	DNN	CGTWDGSLTAGVF
p61.39	p61	IGHV2-5	GFSFSLSGVG	IYWDDDK	CGHHDWGVVGHW	IGKV3-20	QSVISTY	GAT	CQQYDDSRRLTF
p61.40	p61	IGHV3-30	GFTFRNYG	LISYDGSNR	CAKDKAYYYDISGWTFDYW	IGLV3-21	QGINNA	DAS	CQQFSSYPFTF
p61.41	p61	IGHV3-33	GFTFSSYA	ISYDGNNQ	CAREMYYDFWSAYYYAMDVW	IGKV1-27	HDISNY	AAS	CQKYNSAPPLTF
p61.42	p61	IGHV3-49	GFTFADYV	IRSKTYGAAT	CSRAGDFTNFFFDFW	IGLV2-14	SGDIGGYDY	DVT	CTSWSLDTAPYIF
p61.43	p61	IGHV3-66	GFSVTGYY	IYSGGTT	CARGTYTYGYGDIW	IGKV3-20	QSVFSIY	RAS	CQQYGISPFTF
p61.46	p61	IGHV4-34	GGSFSGYY	INQLGNT	CARTINRVGRYFDLW	IGKV1-12	QGLNSW	TTS	CQQANSFPRTF

Grey: Abs isolated from patient 60; Orange: Abs isolated from patient 61

Table S2: Kinetic interaction analysis between nAbs and glycosylated and non-glycosylated GT 3 P domains

**bnAb**	**non-glycosylated GT 3 P domain**		
	**ka (M x s^{-1})**	**kd (s^{-1})**	**KD (M)**
p60.1	$6.87x10^{5}$	$3.3x10^{-5}$	$4.8x10^{-11}$
p60.12	$8.17x10^{4}$	$7.43x10^{-5}$	$9.09x10^{-10}$
p60.15	$3.12x10^{5}$	$5.18x10^{-5}$	$1.66x10^{-10}$
p61.15	$3.14x10^{5}$	$2.56x10^{-5}$	$8.16x10^{-11}$
	**glycosylated GT 3 P domain**		
p60.1	n.d.	n.d.	n.d.
p60.12	n.d.	n.d.	n.d.
p60.15	$5.13x10^{5}$	$1.63x10^{-4}$	$3.18x10^{-10}$
p61.15	$2.41x10^{5}$	$1.21x10^{-4}$	$5.03x10^{-10}$

n.d.: not determined

Table S3: Characteristics of patient samples used for ELISA and isolate neutralization in HLCs

Patient	Sex	Age range	HEV RNA in plasma [10^5 IU/ml]	WANTAI Ag ELISA serum [$OD_{450-630nm}$]	WANTAI IgG ELISA serum [$OD_{450-630nm}$]	Samples used in this study
1	f	60-69	6	3.61	3.36	serum
2	f	30-39	1	3.76	2.89	serum
3	f	40-49	9	4.03	2.62	serum and stool
4	f	30-39	8	3.74	1.72	serum
5	m	50-59	200	3.69	1.93	serum and stool
6	m	70-79	6	2.53	3.58	serum
7	m	50-59	10	3.73	3.67	serum
8	m	50-59	3	3.97	2.19	serum
9	f	30-39	0.9	3.64	0.43	serum and stool
10	f	80-89	8	3.72	n.t.	serum
11	m	60-69	2	>ULOQ	3.29	serum
12	f	40-49	20	3.80	3.04	serum
13	m	40-49	30	3.23	0.01 (negative)	serum and stool
14	f	20-29	70	3.83	0.01 (negative)	serum and stool
60	m	40-49	n.d.	n.d.	3.90	peripheral blood mononuclear cells
61	m	70-79	n.d.	n.d.	>ULOQ	peripheral blood mononuclear cells
378	f	40-49	n.d.	n.d.	3.67	serum
380	f	50-59	n.d.	n.d.	3.83	serum
381	m	60-69	n.d.	n.d.	3.90	serum

IU=international unit; OD= optical density; >ULOQ= above upper limit of quantification

Table S4: Kinetic interaction analysis between nAbs and non-glycosylated P domains of different strains

	HEV GT 1 P domain		
**bnAb**	**ka (M x s^{-1})**	**kd (s^{-1})**	**KD (M)**
p60.1	1.08x10^{6}	2.56x10^{-5}	2.38x10^{-11}
p60.12	9.87x10^{4}	3.25x10^{-4}	3.29x10^{-9}
p60.15	1.78x10^{6}	6.79x10^{-5}	3.81x10^{-11}
p61.15	4.0x10^{5}	6.04x10^{-4}	1.51x10^{-9}
	**HEV GT 2 P domain**		
p60.1	6.36x10^{5}	8.16x10^{-5}	1.28x10^{-10}
p60.12	5.83x10^{4}	2.96x10^{-5}	5.09x10^{-10}
p60.15	1.32x10^{6}	2.3x10^{-4}	1.74x10^{-10}
p61.15	6.53x10^{5}	5.85x10^{-4}	8.96x10^{-10}
	**HEV GT 4 P domain**		
p60.1	1.35x10^{6}	5.14x10^{-5}	3.82x10^{-11}
p60.12	6.96x10^{4}	6.09x10^{-4}	8.75x10^{-9}
p60.15	8.83x10^{5}	1.04x10^{-4}	1.18x10^{-10}
p61.15	9.25x10^{5}	1.42x10^{-4}	1.54x10^{-10}
	**rat HEV P domain**		
p60.1	9.24x10^{4}	4.56x10^{-1}	4.93x10^{-6}
p60.12	4.4x10^{4}	2.46x10^{-1}	5.59x10^{-6}
p60.15	n.d.	n.d.	n.d.
p61.15	n.d.	n.d.	n.d.

n.d.: not determined

Table S5: Diffraction data collection and refinement statistics

	scFv p60.1 + HEV GT3 P domain	Fab p60.12 + HEV GT3 P domain	scFv p60.15 + HEV GT3 P domain	scFv p61.15 + HEV GT3 P domain	Fab p60.12 + rat-HEV P domain
**Data collection**					
Space group	C 2 2 2_1	P 2_1	C 2 2 2_1	P 2_1 2_1 2	P 4_3 2_1 2
Resolution (Å)	43.48 -1.98 (2.05-1.98) [a]	47.24 -2.07 (2.15 -2.07) [a]	48.64 - 2.41 (2.49 - 2.41) [a]	47.85 -1.91 (1.98 -1.91) [a]	48.7 -3.89 (4.031 -3.89) [a]
Cell dimensions					
a, b, c (Å)	75.81 159.26 205.67	88.43 94.48 91.25	56.19 122.95 158.67	108.59 178.77 49.66	112.86 112.86 184.44
α, β, γ (°)	90.0 90.0 90.0	90.0 90.21 90.0	90.0 90.0 90.0	90.0 90.0 90.0	90.0 90.0 90.0
PDB code	8PMW	8PNO	8PMY	8PMZ	8PMX
Complexes in AU	2	2	1	1	1
R_{meas}	0.13 (1.345)	0.129 (1.653)	0.293 (2.45)	0.176 (1.50)	0.322 (4.798)
< I/σ (I) >	12.43 (1.98)	5.3 (0.56)	7.43 (1.01)	11.23 (1.73)	7.68 (0.68)
$CC_{1/2}$	0.998 (0.77)	0.991 (0.193)	0.993 (0.482)	0.997 (0.906)	0.998 (0.401)
Completeness (%)	99.47 (94.93)	84.7 (38.81)	99.61 (99.34)	97.1 (90.4)	99.18 (93.03)
Redundancy	13.23 (21.4)	3.27 (3.8)	13.12 (19.16)	13.52 (25.2)	25.91 (42.45)
**Refinement**					
No. reflections	86310 (8192)	77316 (3527)	21738 (2116)	72647 (5777)	11444
R_{work} / R_{free}	0.191 / 0.211	0.214 / 0.244	0.1993 / 0.238	0.2065 / 0.2395	0.2806 / 0.3537
No. atoms					
Protein	1069	1461	2888	5804	5572
ligands	26	0	3	38	0
solvent	308	184	107	321	0

B-factor					
Protein	45.92	49.31	58.05	36.31	188.35
ligands	60.38		87.37	51.79	
solvent	46.30	35.24	60.94	33.55	
R.m.s deviations					
Bond lenGThs (Å)	0.011	0.011	0.011	0.011	0.010
Bond angles (°)	1.57	1.61	1.63	1.60	1.45
Ramachandran plot[§]					
Favored (%)	96.58	96.04	96.48	97.18	84.09
Allowed (%)	3.23	3.75	3.52	2.82	15.08
Outliers (%)	0.19	0.21	0.00	0.00	0.83
Rotamer outliers (%)	0.67	1.72	1.28	0.00	10.02
Clashscore	0.93	2.63	2.1	1.47	10.74

A single crystal was used to collect each of the individual diffraction data sets

[a] Values in parentheses are for highest-resolution shell.

[§] Ramachandran statistics were calculated with MolProbity

Table S6: Accession numbers of virus strains and isolates used in this study

Synthetic gene	Origin	UniprotKB/ Genbank
HEV ORF2_GT1	isolate/human/Pakistan/Sar55	P33426
HEV ORF2_GT2	isolate/human/Mexico	Q03500
HEV ORF2_GT3	isolate/human/Japan	C4B4T9
HEV ORF2_GT4	isolate/human/China	Q9IVZ8
HEV ORF2_rat	R68/DEU/2009	E0XL23
G3-HEV 83-2-27	Shiota et al., 2013, J Virol	AB740232
Kernow_C1_p6 wt	Isolate/human/United Kingdom	JQ679013

Table S7: HEV antigen detection by different ELISAs

Patient 1, female, aged 60-69:

**timepoint**	**HEV RNA (IU/ml)**	**non-glycosylated pORF2 (p60.1, S/CO)**	**all pORF2 (p60.15, S/CO)**	**WANTAI HEV Ag (S/CO)**
pre-treatment	600000	28,62	30,60	16,45
treatment (TW16)	n. d.	0,75	36,76	19,05
12 months FU	n. d.	0,34	7,69	19,97

Patient 2, male, aged 20-29:

**timepoint**	**HEV RNA (IU/ml)**	**non-glycosylated pORF2 (p60.1, S/CO)**	**all pORF2 (p60.15, S/CO)**	**WANTAI HEV Ag (S/CO)**
pre-treatment	3000000	34,77	29,06	13,60
treatment (TW4)	n. d.	0,88	15,26	13,93
12 months FU	n. d.	0,32	0,57	0,20

Red: HEV detection positive; Green: HEV detection negative; n.d.: not detected; Source data are provided within the Source Data file.

Table S8: List of oligonucleotides used for cloning

**P domain construct**	**Forward primer**	**Reverse primer**
GT 3 P domain_mNeon	CGCTGAAAACCTGTATTTTCAGGGCCCCGCCCCAAGCCGGCCC	GGGTTTAAACTCAGGGCCCTCACGATTCTCGCGTTTTACCC
GT 3 P domain_mRuby	GAGCTGTACAAGGACGATGACGATAAGCCCGCCCCAAGCCGGCCC	GGGTTTAAACTCAGGGCCCTCACGATTCTCGCGTTTTACCC
GT 3 P domain **non-glycosylated**	TTTTCAGGGCGACGATGACGATAAGCCCGCCCCAAGCCGGCCC	GGGTTTAAACTCAGGGCCCTCACGATTCTCGCGTTTTACCC
GT 3 P domain **glycosylated**	TTTTCAGGGCGACGATGACGATAAGCCCGCCCCAAGCCGGCCC	GGGTTTAAACTCAGGGCCCTCACGATTCTCGCGTTTTACCC
GT 1 P domain **non-glycosylated**	TTTTCAGGGCGACGATGACGATAAGCCAGCCCCATCGCGTCCT	GGGTTTAAACTCAGGGCCCTCATAACTCCCGAGTTTTACCCAC
GT 2 P domain **non-glycosylated**	TTTTCAGGGCGACGATGACGATAAGCCAGCCCCCAGCAGGCCA	GGGTTTAAACTCAGGGCCCTCAGAGCTCGCGGGTTTTACC
GT 4 P domain **non-glycosylated**	TTTTCAGGGCGACGATGACGATAAGCCCGCCCCCAGTCGCCCG	GGGTTTAAACTCAGGGCCCTCAGTATTCACGTGTCTTGCCCA
rat HEV P domain **non-glycosylated**	TTTTCAGGGCGACGATGACGATAAGCCCGCACCTGCCCGACCT	GGGTTTAAACTCAGGGCCCTCATACGCTATCGGCTGCGGC
**IgG construct**	**Forward primer**	**Reverse primer**
p60.1-HC	GCTGGTCGCTTCCTGCCTGGGCGAAGTTCAGCTGCAGCAGA	CCGATGGGCCCTTCGTACTGGCCGAACTAACAGTGACCAACG
p60.1-KC	GCTGGTCGCTTCCTGCCTGGGCATTCAGATGACACAAAGCCCT	CGGATGGGGCGGCCACGGTGCGCTTCAAATCGACTTTTGTTCCCG
p60.4-HC	GCTGGTCGCTTCCTGCCTGGGCCAGGTACAGCTGGTACAAAGC	CCGATGGGCCCTTCGTACTGGCACTCGATACCGTTACTGTAGTTC
p60.4-KC	GCTGGTCGCTTCCTGCCTGGGCGATATACAAATGACACAGAGCCCG	CGGATGGGGCGGCCACGGTGCGTTTTATCTCCACCTTAGTACCTCCT

p60.5-HC	GCTGGTCGCTTCCTGCCTGGGCGAAGTTCAACTCGTGGAAAGTG	CCGATGGGCCCTTCGTACTGGCACTACTCACCGTCACTGTAGT
p60.5-KC	GCTGGTCGCTTCCTGCCTGGGCGATATTGTGATGACTCAGAGCCC	CGGATGGGGCGGCCACGGTGCGTTTAATTTCGAGCTTGGTGCC
p60.6-HC	GCTGGTCGCTTCCTGCCTGGGCGAGGTTCAACTGCTGGAAAGT	CCGATGGGCCCTTCGTACTGGCGCTGGATACCGTTACCAAAGT
p60.6-LC	GCTGGTCGCTTCCTGCCTGGGCCAGTCCGCATTGACTCAAC	ATGGGGCGGCCTTCGGCTGGCCCAGGACTGTGAGTTTGGTGC
p60.10-HC	GCTGGTCGCTTCCTGCCTGGGCCAGGTGCAACTCCAGCAA	CCGATGGGCCCTTCGTACTGGCCGACGATACAGTTACCAAAGTG
p60.10-LC	GCTGGTCGCTTCCTGCCTGGGCCAAAGTGCTTTGACGCAACC	ATGGGGCGGCCTTCGGCTGGCCCAGGACTGTCACCTTCGT
p60.12-HC	GCTGGTCGCTTCCTGCCTGGGCCAGGTACAGTTGGTTCAAAGTG	CCGATGGGCCCTTCGTACTGGCGCTGCTGACCGTCACCAA
p60.12-LC	GCTGGTCGCTTCCTGCCTGGGCCAGAGTGCTCTCACTCAACC	ATGGGGCGGCCTTCGGCTGGCCGAGAACAGTGAGCTTGGTGC
p60.13-HC	GCTGGTCGCTTCCTGCCTGGGCGAAGTGCAGCTCGTTGAGT	CCGATGGGCCCTTCGTACTGGCCGAGGAGACTGTTACGAGAG
p60.13-LC	GCTGGTCGCTTCCTGCCTGGGCCAATCCGTTTTGACTCAACCC	ATGGGGCGGCCTTCGGCTGGCCGAGAACAGTGAGCTTAGTTCCA
p60.15-HC	GCTGGTCGCTTCCTGCCTGGGCGAAGTCCAACTCGTGCAATC	CCGATGGGCCCTTCGTACTGGCCGAGCTAACAGTAACCAATGTTC
p60.15-KC	GCTGGTCGCTTCCTGCCTGGGCGCGATACAATTGACACAGAGTC	CGGATGGGGCGGCCACGGTGCGTTTGATATCGACCTTTGTTCCAGG
p60.17-HC	GCTGGTCGCTTCCTGCCTGGGCCAAGTCCAACTCGTTCAGTCC	CCGATGGGCCCTTCGTACTGGCCGAACTAACCGTTACGAGCG
p60.17-LC	GCTGGTCGCTTCCTGCCTGGGCCAGTCGGCACTCACTCAA	ATGGGGCGGCCTTCGGCTGGCCCAAGACGGTCAATTTCGTTCC
p60.26-HC	GCTGGTCGCTTCCTGCCTGGGCCAAGTGCAATTGGTGCAATCG	CCGATGGGCCCTTCGTACTGGCGCTCGACACGGTTACGGT
p60.26-LC	GCTGGTCGCTTCCTGCCTGGGCTCCTATGAACTGACTCAACCAC	ATGGGGCGGCCTTCGGCTGGCCCAGAACTGTCAGCTTGGTGC
p60.34-HC	GCTGGTCGCTTCCTGCCTGGGCCAAGTGCAATTGGTGCAGT	CCGATGGGCCCTTCGTACTGGCGCTGCTTACCGTTACCAGC
p60.34-LC	GCTGGTCGCTTCCTGCCTGGGCCAGCTGGTTCTCACGCAA	ATGGGGCGGCCTTCGGCTGGCCCAAGACGGTCAGTTTTGTGC
p60.35-HC	GCTGGTCGCTTCCTGCCTGGGCCAAGTACATTTGGTGCAGTCG	CCGATGGGCCCTTCGTACTGGCGGAGGATATGGTCACGAGGG
p60.35-LC	GCTGGTCGCTTCCTGCCTGGGCAATTTTATGTTGACACAGCCTCATA	ATGGGGCGGCCTTCGGCTGGCCCAGTACCGTGAGTTTTGTGC
p60.36-HC	GCTGGTCGCTTCCTGCCTGGGCCAGGTGCAGTTGGTTCAAAG	CCGATGGGCCCTTCGTACTGGCCGAGCTCACGGTTACCAA
p60.36-LC	GCTGGTCGCTTCCTGCCTGGGCAGCTATGAATTGACTCAACCACC	ATGGGGCGGCCTTCGGCTGGCCCAGGACCGTCAGCTTGGT
p60.45-HC	GCTGGTCGCTTCCTGCCTGGGCCAAGTACAGCTCGTGCAGT	CCGATGGGCCCTTCGTACTGGCGCTGCTTACCGTAACCAATG
p60.45-LC	GCTGGTCGCTTCCTGCCTGGGCAGCTACGTTCTGACACAACC	ATGGGGCGGCCTTCGGCTGGCCCAGTGCTGTCAGTTTAGTGCC
p61.15-HC	GCTGGTCGCTTCCTGCCTGGGCCAAGTGCAACTCGTGCAAT	CCGATGGGCCCTTCGTACTGGCACTACTCACGGTAACGAGAGT
p61.15-KC	GCTGGTCGCTTCCTGCCTGGGCGAAATAGTTCTCACACAGTCCCC	CGGATGGGGCGGCCACGGTGCGTTTTATTTCGAGCCTTGTTCCTTG
8G12-HC	GCTGGTCGCTTCCTGCCTGGGCGGACAGCTGCAGCAGAGT	CCGATGGGCCCTTCGTACTGGCACTCGATACTGTAACCAACGTG
8G12-KC	GCTGGTCGCTTCCTGCCTGGGCGACATACAAATGACTCAGAGTCCT	CGGATGGGGCGGCCACGGTGCGCTTGATTTCCAATTTTGTACCGCT

Source Data for Fig. S1: HEV antigen detection by different ELISAs

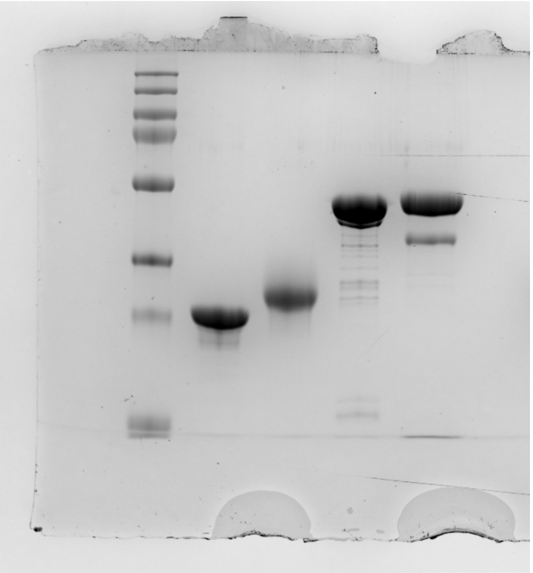

Supplement: Supplementary file 1 — Supplementary Information [file 41467_2025_57182_MOESM1_ESM.pdf]
