# Supplementary material for: Broadly neutralizing antibodies isolated from HEV convalescents confer protective effects in human liver-chimeric mice
Source: Nat Commun. 2025 Feb 26;16:1995. doi: 10.1038/s41467-025-57182-1 (PMC11865592; doi:10.1038/s41467-025-57182-1)
Supplement: Supplementary file 2 — Reporting Summary [file 41467_2025_57182_MOESM2_ESM.pdf]

nature portfolio

Corresponding author(s): Thomas Krey

Last updated by author(s): Oct 28, 2023

Reporting Summary

Nature Portfolio wishes to improve the reproducibility of the work that we publish. This form provides structure for consistency and transparency in reporting. For further information on Nature Portfolio policies, see our Editorial Policies and the Editorial Policy Checklist.

Statistics

For all statistical analyses, confirm that the following items are present in the figure legend, table legend, main text, or Methods section.

n/a	Confirmed	
☐	☒	The exact sample size (*n*) for each experimental group/condition, given as a discrete number and unit of measurement
☐	☒	A statement on whether measurements were taken from distinct samples or whether the same sample was measured repeatedly
☒	☐	The statistical test(s) used AND whether they are one- or two-sided<br>*Only common tests should be described solely by name; describe more complex techniques in the Methods section.*
☒	☐	A description of all covariates tested
☒	☐	A description of any assumptions or corrections, such as tests of normality and adjustment for multiple comparisons
☐	☒	A full description of the statistical parameters including central tendency (e.g. means) or other basic estimates (e.g. regression coefficient) AND variation (e.g. standard deviation) or associated estimates of uncertainty (e.g. confidence intervals)
☒	☐	For null hypothesis testing, the test statistic (e.g. *F*, *t*, *r*) with confidence intervals, effect sizes, degrees of freedom and *P* value noted<br>*Give P values as exact values whenever suitable.*
☒	☐	For Bayesian analysis, information on the choice of priors and Markov chain Monte Carlo settings
☒	☐	For hierarchical and complex designs, identification of the appropriate level for tests and full reporting of outcomes
☒	☐	Estimates of effect sizes (e.g. Cohen's *d*, Pearson's *r*), indicating how they were calculated

Our web collection on statistics for biologists contains articles on many of the points above.

Software and code

Policy information about availability of computer code

Data collection	*Provide a description of all commercial, open source and custom code used to collect the data in this study, specifying the version used OR state that no software was used.*
Data analysis	X-ray data collection, processing, analysis and representation used the following software packages:<br>XDS (version 2022), programs from the CCP4 8.0 suite, Phaser 2.8.3, Coot 0.9.7, AutoBuster 2.10.4, MolProbity, PYMOL 2.5<br>Infection of HLCs was analysed using Zen software.<br>Flow cytometry analysis of single B cells was analysed using the FCS Express 7 software, sequencing data was analyzed with the Single Cell Immune Profiling Solution (10x Genomics) and analysed using the Loupe V(D)J Browser 3.0.0. Productive sequences were re-annotated with IMGT/HighV-Quest.<br>Surface Plasmon resonance results were analysed using the Biacore X100 Evaluation software.<br>ELISA and neutralisation data were analyzed using GraphPad prism 9.

For manuscripts utilizing custom algorithms or software that are central to the research but not yet described in published literature, software must be made available to editors and reviewers. We strongly encourage code deposition in a community repository (e.g. GitHub). See the Nature Portfolio guidelines for submitting code & software for further information.

Data

Policy information about availability of data

All manuscripts must include a data availability statement. This statement should provide the following information, where applicable:

- Accession codes, unique identifiers, or web links for publicly available datasets
- A description of any restrictions on data availability
- For clinical datasets or third party data, please ensure that the statement adheres to our policy

The atomic coordinates and structure factors for five crystal structures in this study were deposited in the Protein Data Bank (http://www.pdb.org/) under the accession numbers 8PMW, 8PMX, 8PMY, 8PMZ, and 8PN0. The scRNAseq data were deposited at the European Nucleotide Archive (PRJEB76306) and the Sequence Read Archive (PRJNA1166121). All raw data generated in this study are provided in the supplementary information or the Source Data file.

Research involving human participants, their data, or biological material

Policy information about studies with human participants or human data. See also policy information about sex, gender (identity/presentation), and sexual orientation and race, ethnicity and racism.

Reporting on sex and gender	The patient's sex was determined based on self-report and not considered in the selection of patients. Overall, 9 female and 10 male patients were included.
Reporting on race, ethnicity, or other socially relevant groupings	Patients included in this study were of caucasian ethnicity.
Population characteristics	The patients included in this study were between the age of 21 and 79 at study participation. Patients with self-resolving HEV infection had no other relevant diagnosis. Patients with chronic HEV infection were immunosuppressed due to solid organ transplantation.
Recruitment	The participating individuals gave written informed consent to the study and utilization of the biomaterial and information on the course of HEV infection. They were recruited between April 1st, 2019 and February 1st, 2022.
Ethics oversight	This study was conducted in accordance with the Declaration of Helsinki and is approved by the local Ethics committee (No. 8743_BO_K_2019).

Note that full information on the approval of the study protocol must also be provided in the manuscript.

Field-specific reporting

Please select the one below that is the best fit for your research. If you are not sure, read the appropriate sections before making your selection.

☒ Life sciences ☐ Behavioural & social sciences ☐ Ecological, evolutionary & environmental sciences

For a reference copy of the document with all sections, see nature.com/documents/nr-reporting-summary-flat.pdf

Life sciences study design

All studies must disclose on these points even when the disclosure is negative.

Sample size	N/A
Data exclusions	No data were excluded.
Replication	Neutralization and ELISA experiments were at least performed in two biological replicates each with technical replicates wherever possible.
Randomization	In all experiments, organisms were allocated randomly to the experimental groups.
Blinding	Investigators were not blinded to group allocation.

Behavioural & social sciences study design

All studies must disclose on these points even when the disclosure is negative.

Study description	*Briefly describe the study type including whether data are quantitative, qualitative, or mixed-methods (e.g. qualitative cross-sectional, quantitative experimental, mixed-methods case study).*

Research sample	*State the research sample (e.g. Harvard university undergraduates, villagers in rural India) and provide relevant demographic information (e.g. age, sex) and indicate whether the sample is representative. Provide a rationale for the study sample chosen. For studies involving existing datasets, please describe the dataset and source.*
Sampling strategy	*Describe the sampling procedure (e.g. random, snowball, stratified, convenience). Describe the statistical methods that were used to predetermine sample size OR if no sample-size calculation was performed, describe how sample sizes were chosen and provide a rationale for why these sample sizes are sufficient. For qualitative data, please indicate whether data saturation was considered, and what criteria were used to decide that no further sampling was needed.*
Data collection	*Provide details about the data collection procedure, including the instruments or devices used to record the data (e.g. pen and paper, computer, eye tracker, video or audio equipment) whether anyone was present besides the participant(s) and the researcher, and whether the researcher was blind to experimental condition and/or the study hypothesis during data collection.*
Timing	*Indicate the start and stop dates of data collection. If there is a gap between collection periods, state the dates for each sample cohort.*
Data exclusions	*If no data were excluded from the analyses, state so OR if data were excluded, provide the exact number of exclusions and the rationale behind them, indicating whether exclusion criteria were pre-established.*
Non-participation	*State how many participants dropped out/declined participation and the reason(s) given OR provide response rate OR state that no participants dropped out/declined participation.*
Randomization	*If participants were not allocated into experimental groups, state so OR describe how participants were allocated to groups, and if allocation was not random, describe how covariates were controlled.*

Ecological, evolutionary & environmental sciences study design

All studies must disclose on these points even when the disclosure is negative.

Study description	*Briefly describe the study. For quantitative data include treatment factors and interactions, design structure (e.g. factorial, nested, hierarchical), nature and number of experimental units and replicates.*
Research sample	*Describe the research sample (e.g. a group of tagged Passer domesticus, all Stenocereus thurberi within Organ Pipe Cactus National Monument), and provide a rationale for the sample choice. When relevant, describe the organism taxa, source, sex, age range and any manipulations. State what population the sample is meant to represent when applicable. For studies involving existing datasets, describe the data and its source.*
Sampling strategy	*Note the sampling procedure. Describe the statistical methods that were used to predetermine sample size OR if no sample-size calculation was performed, describe how sample sizes were chosen and provide a rationale for why these sample sizes are sufficient.*
Data collection	*Describe the data collection procedure, including who recorded the data and how.*
Timing and spatial scale	*Indicate the start and stop dates of data collection, noting the frequency and periodicity of sampling and providing a rationale for these choices. If there is a gap between collection periods, state the dates for each sample cohort. Specify the spatial scale from which the data are taken*
Data exclusions	*If no data were excluded from the analyses, state so OR if data were excluded, describe the exclusions and the rationale behind them, indicating whether exclusion criteria were pre-established.*
Reproducibility	*Describe the measures taken to verify the reproducibility of experimental findings. For each experiment, note whether any attempts to repeat the experiment failed OR state that all attempts to repeat the experiment were successful.*
Randomization	*Describe how samples/organisms/participants were allocated into groups. If allocation was not random, describe how covariates were controlled. If this is not relevant to your study, explain why.*
Blinding	*Describe the extent of blinding used during data acquisition and analysis. If blinding was not possible, describe why OR explain why blinding was not relevant to your study.*

Did the study involve field work? ☐ Yes ☐ No

Field work, collection and transport

Field conditions	*Describe the study conditions for field work, providing relevant parameters (e.g. temperature, rainfall).*
Location	*State the location of the sampling or experiment, providing relevant parameters (e.g. latitude and longitude, elevation, water depth).*
Access & import/export	*Describe the efforts you have made to access habitats and to collect and import/export your samples in a responsible manner and in compliance with local, national and international laws, noting any permits that were obtained (give the name of the issuing authority,*

the date of issue, and any identifying information).

Disturbance | *Describe any disturbance caused by the study and how it was minimized.*

Reporting for specific materials, systems and methods

We require information from authors about some types of materials, experimental systems and methods used in many studies. Here, indicate whether each material, system or method listed is relevant to your study. If you are not sure if a list item applies to your research, read the appropriate section before selecting a response.

Materials & experimental systems

n/a	Involved in the study
☐	☒ Antibodies
☐	☒ Eukaryotic cell lines
☒	☐ Palaeontology and archaeology
☐	☒ Animals and other organisms
☐	☒ Clinical data
☒	☐ Dual use research of concern
☒	☐ Plants

Methods

n/a	Involved in the study
☒	☐ ChIP-seq
☐	☒ Flow cytometry
☒	☐ MRI-based neuroimaging

Antibodies

Antibodies used

- anti-pORF2 rabbit polyclonal IgG was isolated in the course of this study from a rabbit immunized twice with the P domain of pORF-2 (pGS99) by Davids Biotechnology, Regensburg, Germany. The serum was used in a 1:5,000 dilution
- horseradish peroxidase-conjugated goat anti-rabbit antibody (Invitrogen, reference: 31460, LOT: WC320195) diluted 1:10,000
- Alexa Fluor™ 488, goat anti-human (Invitrogen, reference: A-11013), 1:1,000
- Alexa Fluor™ 488, goat anti-human antibody (Invitrogen, reference: A-11013), 1:1,000
- HEV pORF3 specific antibody (1:1600, reference: bs-0212R, Bioss, Woburn, USA)
- Cytokeratin 18 (DC-10) antibody (1:400, reference: sc-6259, Santa Cruz, Heidelberg, Germany)

Validation

The serum of the rabbit was tested in initial validation experiments for the specificity to bind HEV ORF-2 protein in infected cells.

Eukaryotic cell lines

Policy information about cell lines and Sex and Gender in Research

Cell line source(s)

Mammalian cell-lines were obtained from ATCC (HepG2: HB-8065; HepG2/C3A: CRL-10741). Drosophila melanogaster Schneider2 cells were obtained from Life Technologies (https://www.thermofisher.com/order/catalog/product/de/de/R69007). The iPSC cell line was generated by Stephen Duncan (https://aasldpubs.onlinelibrary.wiley.com/doi/10.1002/hep.23354), the donor was male.

Authentication

For mammalian cell lines authentication was provided by ATCC and checked by in house RNA-Sequencing data. For Drosophila melanogaster Schneider 2 cells no authentication was performed.

Mycoplasma contamination

All cell lines were regularly tested negative for Mycoplasma contamination. Drosophila melanogaster Schneider2 cells were not regularly tested for mycoplasma contamination.

Commonly misidentified lines
(See ICLAC register)

No commonly misidentified cell lines were used in this study.

Animals and other research organisms

Policy information about studies involving animals; ARRIVE guidelines recommended for reporting animal research, and Sex and Gender in Research

Laboratory animals

Homozygous uPA+/+-SCID mice and uPA/SCID/beige/IL2rg-/-(USG) mice were transplanted with approximately 1.000.000 primary human hepatocytes to obtain human liver chimeric mice.

Wild animals

Provide details on animals observed in or captured in the field; report species and age where possible. Describe how animals were caught and transported and what happened to captive animals after the study (if killed, explain why and describe method; if released, say where and when) OR state that the study did not involve wild animals.

Reporting on sex

Both male and female mice were used in this study

Field-collected samples

For laboratory work with field-collected samples, describe all relevant parameters such as housing, maintenance, temperature, photoperiod and end-of-experiment protocol OR state that the study did not involve samples collected from the field.

Ethics oversight | The HEV GT3 animal experiments performed in Ghent were in accordance with the European Communities Council Directive (86/609/EEC) and approved by the Animal Ethics Committee of the Faculty of Medicine and Health Sciences, Ghent University (refs. ECD17-93 and ECD19-74). All HEV GT1 animal experiments were conducted in accordance with the European Communities Council Directive (86/609/EEC) and were approved by the City of Hamburg, Germany.

Note that full information on the approval of the study protocol must also be provided in the manuscript.

Clinical data

Policy information about clinical studies

All manuscripts should comply with the ICMJEguidelines for publication of clinical research and a completedCONSORT checklist must be included with all submissions.

Clinical trial registration | *Provide the trial registration number from ClinicalTrials.gov or an equivalent agency.*

Study protocol | *Note where the full trial protocol can be accessed OR if not available, explain why.*

Data collection | *Describe the settings and locales of data collection, noting the time periods of recruitment and data collection.*

Outcomes | *Describe how you pre-defined primary and secondary outcome measures and how you assessed these measures.*

Plants

Seed stocks | *Report on the source of all seed stocks or other plant material used. If applicable, state the seed stock centre and catalogue number. If plant specimens were collected from the field, describe the collection location, date and sampling procedures.*

Novel plant genotypes | *Describe the methods by which all novel plant genotypes were produced. This includes those generated by transgenic approaches, gene editing, chemical/radiation-based mutagenesis and hybridization. For transgenic lines, describe the transformation method, the number of independent lines analyzed and the generation upon which experiments were performed. For gene-edited lines, describe the editor used, the endogenous sequence targeted for editing, the targeting guide RNA sequence (if applicable) and how the editor was applied.*

Authentication | *Describe any authentication procedures for each seed stock used or novel genotype generated. Describe any experiments used to assess the effect of a mutation and, where applicable, how potential secondary effects (e.g. second site T-DNA insertions, mosiacism, off-target gene editing) were examined.*

Flow Cytometry

Plots

Confirm that:

☐ The axis labels state the marker and fluorochrome used (e.g. CD4-FITC).

☐ The axis scales are clearly visible. Include numbers along axes only for bottom left plot of group (a 'group' is an analysis of identical markers).

☐ All plots are contour plots with outliers or pseudocolor plots.

☐ A numerical value for number of cells or percentage (with statistics) is provided.

Methodology

Sample preparation | Frozen PBMCs from the two donors were thawed on ice and resuspended in MACS buffer (PBS pH 7.2, 2 mM EDTA, 0.5% (w/v) BSA) after written consent by the participants. Cells were pelleted (350 x g, 4 °C, 10 min) and resuspended in 80 µl buffer and 20 µl human CD19 microbeads (Miltenyi Biotec) per 107 cells. After a 15-minute incubation at 4 °C, cells were pelleted, followed by resuspension of up to 108 cells in 500 µl MACS buffer for magnetic cell separation on LS columns (Miltenyi Biotec) per the manufacturer's instructions. The MACs-sorted cells were labeled (0.2 mg/ml GT3-mNeon-fused P domain (non-glycosylated), 0.2 mg/ml GT3-mRuby-fused P domain (glycosylated), 20 µl APC Mouse Anti Human IgG (BD Bioscience), and 5 µL Alexa Fluor 700 Mouse Anti-Human CD20 (BD Biosciences) per 106 cells in 100 µL, in addition to LIVE/DEAD™ Fixable Near-IR Dead Cell Stain (ThermoFisher Scientific) (1 µl stain in 1 ml)), incubated for 30 min on ice, and later washed with MACS buffer prior to resuspension in 400 µl PBS supplemented with 0.5% (w/v) BSA.

Instrument | Cells were sorted on a BD Bioscience FACSAria III Fusion sorter.

Software | Flow cytometry analysis of single B cells was analysed using the FCS Express 7 software.

Cell population abundance | *Describe the abundance of the relevant cell populations within post-sort fractions, providing details on the purity of the samples and how it was determined.*

Gating strategy

Describe the gating strategy used for all relevant experiments, specifying the preliminary FSC/SSC gates of the starting cell population, indicating where boundaries between "positive" and "negative" staining cell populations are defined.

☒ Tick this box to confirm that a figure exemplifying the gating strategy is provided in the Supplementary Information.
